# Thermal Properties and Fracture Toughness of Epoxy Nanocomposites Loaded with Hyperbranched-Polymers-Based Core/Shell Nanoparticles

**DOI:** 10.3390/nano9030418

**Published:** 2019-03-12

**Authors:** Aldobenedetto Zotti, Simona Zuppolini, Anna Borriello, Mauro Zarrelli

**Affiliations:** Institute for Polymers, Composites and Biomaterials, National Research Council of Italy, 80055 Portici, Naples, Italy; aldobenedetto.zotti@unina.it (A.Z.); simona.zuppolini@cnr.it (S.Z.); mauro.zarrelli@cnr.it (M.Z.)

**Keywords:** epoxy resin, core/shell nanoparticles (CSNPs), polymer-matrix composites (PMCs), thermal stability, fracture toughness

## Abstract

Synthesized silicon oxide (silica) nanoparticles were functionalized with a hyperbranched polymer (HBP) achieving a core/shell nanoparticles (CSNPs) morphology. CSNPs were characterized by Fourier Transform Infrared (FTIR) spectroscopy, Transmission Electron Microscopy (TEM), and Thermogravimetric Analysis (TGA). A core diameter of about 250 nm with a 15 nm thick shell was revealed using TEM images. An aeronautical epoxy resin was loaded with the synthesized CSNPs at different percentages and thermal properties, such as thermal stability and dynamic mechanical properties, were investigated with the use of different techniques. Although the incorporation of 2.5 wt% of CSNPs induces a ~4 °C reduction of the hosting matrix glass transition temperature, a slight increase of the storage modulus of about ~10% was also measured. The Kissinger Method was employed in order to study the thermal stability of the nanocomposites; the degradation activation energies that resulted were higher for the sample loaded with low filler content with a maximum increase of both degradation step energies of about ~77% and ~20%, respectively. Finally, fracture toughness analysis revealed that both the critical stress intensity factor (K_IC_) and critical strain energy release rate (G_IC_) increased with the CSNPs content, reporting an increase of about 32% and 74%, respectively, for the higher filler loading.

## 1. Introduction

Epoxy resins are characterized by many advantages including good mechanical properties, low specific weight compared to metals, and high corrosion resistance. These characteristics have made epoxy resins the main thermoset materials commercially, especially as high-performance adhesives and a composite materials matrix [1,2]. However, these resins are characterized by low thermal stability and fire resistance, and they can produce toxic smokes during combustion processes [3]. Moreover, the intrinsic brittleness of epoxy resins limits their use in some advanced applications for which high toughness is required, such as adhesives and fiber-reinforced polymers for aerospace applications [4]. For these reasons, epoxy resins are generally loaded with different filler typologies, such as inorganic materials, core/shell nanoparticles [5], and hyperbranched polymers (HBP) [6].

Inorganic fillers are generally used in thermosetting resins in order to reduce the coefficient of the thermal expansion of finished products and to increase the thermal stability of the composite system. In particular, silica [7] and inorganic clays (e.g., montmorillonite [8] and sepiolite [9]) are the most used inorganic fillers to improve the thermal and mechanical properties of epoxy resins. For example, Tarrío-Saavedra et al. [10] reported the increase of thermal stability and the reduction of the mass loss rate by the addition of fumed silica to an epoxy matrix. A significant reduction of the PHRR (Peak of Heat Release Rate) was observed by Zotti et al. [11] by adding 10% by weight of sepiolite, and, therefore, a remarkable improvement of the hosting epoxy matrix fire behavior and thermal stability was achieved. Additionally, Nazir et al. [12] have demonstrated an enhancement of the epoxy thermal stability with the incorporation of silica; in particular, all the temperatures of degradation, i.e., Td^10^ (10% weight loss), Td^50^ (50% weight loss), and T^max^ (maximum weight loss), have increased with the growth in silica domains of the hybrid epoxy–silica polymer. Inorganic fillers are also widely employed as tougheners in the epoxy matrix; Ragosta et al. [13] have demonstrated that the addition of silica nanoparticles up to 10 wt% causes a remarkable enhancement in the fracture toughness and an increase in the critical crack length for the onset of crack propagation. Besides these reported benefits, inorganic nanoparticles can lead to a significant increase of the resin viscosity due to their high specific surface area, which inevitably will detrimentally affect the level processability of the hosting system in manufacturing composite materials and structures.

HBP strongly increase the fracture toughness and impact strength of the hosting matrix [6] but, on the other hand, they can reduce the thermomechanical properties. This is the case reported by Fu et al. [14] who have synthesized a hyperbranched poly(trimellitic anhydride-triethylene glycol) ester epoxy (HTTE) to toughen a diglycidyl ether of bisphenol-A (DGEBA) epoxy system. Reported experimental data reveal that the glass transition temperature (T_g_) and the thermal stability decrease to some extent compared with those of the neat epoxy, but at the same time, the impact strength is improved by two to seven times. Improvements in the critical stress intensity factor (K_IC_) and impact strength without loss of the thermomechanical properties can be observed in nanocomposites loaded with HBP, as reported by Jin et al. [15]. In this work, the K_IC_ value and impact strength are 2.5 and 2 times the values of the neat epoxy resins, respectively, without any loss in the thermal stability and with an increase of T_g_ of about 6 °C compared to that of neat DGEBA.

Lately, the addition of core/shell particles to epoxy resins has proven to be a good solution to solve thermal stability and toughness problems. Employing core/shell rubber (CSR) particles to modify a low crosslink density epoxy resin, Sue et al. [16] have obtained an increase of K_IC_ and G_IC_ of about 232% and 455%, respectively, with a slight reduction of elastic modulus and without variations of the glass transition temperature. Quan et al. [17] have demonstrated that smaller CSR particles are more effective than the bigger ones in increasing the thermal stability of the epoxy matrix, with an increase of T_g_ up to 9 °C. Furthermore, in this case, the authors report a considerable increase in K_IC_ and G_IC_ values. Core/shell particles are not used only for the purpose of increasing hosting matrix thermal stability or fracture toughness, but they are also employed to confer specific characteristics to the matrix, such as magnetic [18] and dielectric [19] properties. For example, Huang et al. [16] have incorporated silica core/RGO (Reduced Graphene Oxide) shell nanoparticles in an epoxy matrix in order to increase its dielectric constant and thermal conductivity. The silica core was used to improve the graphene dispersion and obtain epoxy composites with improved thermo-mechanical properties.

The focus of this work was to improve the thermal stability and fracture toughness of an aeronautical-grade epoxy resin by adding core/shell nanoparticles (CSNPs) constituted by a silica core and HBP shell. The HBP were grown on the modified surface of silica nanoparticles, through polycondensation of succinic anhydride and diethanolamine. The potential enhancement of both the thermal stability and mechanical properties of the corresponding nanocomposite systems were examined by different experimental methods, i.e., thermogravimetric analysis (TGA), dynamic mechanical analysis (DMA), and fracture toughness tests, in order to investigate the potential enhancement of both the thermal stability and mechanical property.

Data reveal that loading the hosting system with synthesized nanofillers will enhance the thermal stability of the final compound with a maximum achieved increase of ~77% and ~20%, respectively, for the first and second degradation step. The filler concentration also affects the fracture toughness of the final compounds with an improved value of about 32% and 74% at higher filler loading, respectively, for the critical stress intensity factor and critical strain energy release rate.

## 2. Materials and Methods

### 2.1. Materials

Tetraethyl orthosilicate (TEOS), (3-aminopropyl)triethoxysilane (APTES), succinic anhydride, diethanolamine (DEA), dimethylformamide (DMF) and all solvents were purchased from Sigma-Aldrich. All the chemicals were used as received.

The used resin system was RTM6 epoxy supplied by Hexcel Composites (Duxford, UK); this is a pre-mixed epoxy-amine system developed for liquid infusion processes as characterized by a very low viscosity (~50 mPa·s at 120 °C).

### 2.2. CSNPs Synthesis

The CSNPs preparation consisted of two steps: The synthesis of the amino-functionalized silica core and the HBP grown on the surface. The amino-functionalized silica nanoparticles (NPs) were prepared using a modified Stöber method [20] starting from TEOS and APTES as silica precursors. An equimolar mixture of TEOS (9.8 mL) and APTES (10.3 mL) was added dropwise to a solution of ethanol (50 mL), water (18 mL), and ammonia (6.3 mL) with stirring, and the system was heated under reflux at 78 °C for 60 min. The resulting solution was filtered, washed with deionized water, and dried in an oven at 90 °C under vacuum overnight.

**Figure 1:**
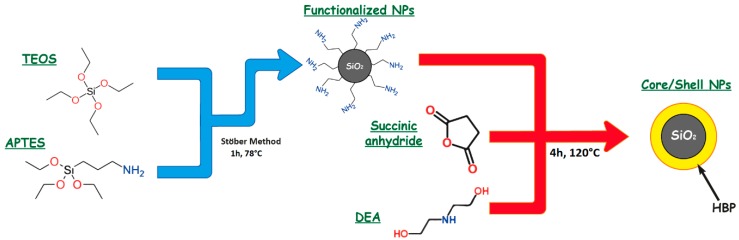
The synthesis route of core/shell nanoparticles (CSNPs).

The HBP shell was grown on the silica surface by polycondensation of succinic anhydride and DEA, according to a modified method reported in the literature [21]. The previously synthesized silica NPs (1.4 g) were mixed with succinic anhydride (3.14 g) and the reaction was conducted under magnetic stirring, in a nitrogen atmosphere at 140 °C for 4 h. Then, DEA (2.74 mL) was added to the mixture under a nitrogen atmosphere at 120 °C for 4 h. Afterward, the system was vacuumed for 2 h and the crude product was dissolved in DMF and centrifuged for 10 min to remove pure hyperbranched poly-amide esters. Finally, the obtained CSNPs were dried at 80 °C under vacuum overnight. The CSNPs fabrication route is reported in the scheme reported in Figure 1.

### 2.3. Nanocomposites Preparation

**Figure 2:**
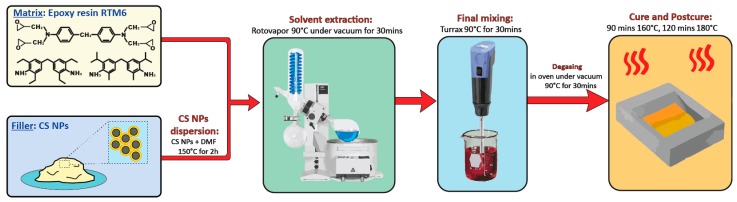
The manufacturing procedure of CSNPs/RTM6 nanocomposites.

To prepare the CSNPs/RTM6 nanocomposites, the appropriate amount (1 wt% and 2.5 wt%) of CSNPs was dissolved in 10 mL of DMF at 150 °C for 1 h. Then, the solution was mixed with the epoxy resin in a round-bottom flask and the solvent was removed using a rotavapor (at 90 °C under vacuum). The solvent-free mixture was mechanically mixed using a Dispersing System ULTRA-TURRAX^®^ T 25 digital at 90 °C for 30 min and then degassed in an oven under vacuum at 90 °C for 1 h. The modified epoxy system was poured into stainless steel templates, previously coated with a release agent (FREKOTE 70), and then cured using the RTM6 standard curing cycle [18], i.e., 90 min at 160 °C, followed by a post-curing stage of 2 h at 180 °C. The cured plates were slowly cooled down to room temperature and cut to prescribed sample dimensions according to the test’s standards. The CSNPs/RTM6 nanocomposites manufacturing procedure is reported schematically in Figure 2.

### 2.4. Characterization

A Fourier transform infrared spectrum (FTIR) was recorded on a Perkin Elmer Spectrum 100 FTIR spectrophotometer (Milano, Italy) in the 4000–400 cm^−1^ region. Scanning electron microscopy (SEM) images were recorded using an FEI Quanta 200 FEG, while Bright field transmission electron microscopy (TEM) analysis was performed by means of an FEI Tecnai G12 Spirit Twin, equipped with a LaB6 source and a FEI Eagle 4k CCD camera (Eindhoven, The Netherlands).

The calorimetric analysis of the manufactured nanocomposites was carried out by using a Differential Scanning Calorimeter (DSC) Q1000 by TA Instruments under a nitrogen atmosphere (50 mL/min) and a heating ramp of 10 °C/min with a sample weight of 5 ± 0.5 mg. A TA Instruments Q500 system was employed for thermogravimetrical analysis (TGA); tests were conducted in an air atmosphere (50 mL/min) and using a heating ramp equal to 2, 5, or 10 °C/min. A sample weight of around 5 ± 0.5 mg was considered for each run test from ambient temperature to 700 °C.

Dynamic mechanical analysis (DMA) was performed by using the Q800 system (DMA) by TA Instruments. The testing configuration was the double cantilever mode, with the nominal sample dimension of 60 × 12 × 2.5 mm and a heating ramp of 3 °C/min; all tests were performed by setting 60 μm as the amplitude and 1 Hz as the frequency. For thermal (DSC and TGA) and thermo-mechanical (DMA) properties, three tests for each sample were performed.

A single edge notched beam (SENB) configuration was employed for Mode-I fracture tests. The test specimen was chosen according to the ASTM D5045-99 standard test method for the plane strain fracture toughness and strain energy release rate of plastic materials. The plane strain condition was obtained using samples with the following dimensions: 3 mm for the thickness (B), 6 mm for the width (W), and 24 mm for the supporting span (S). The crack length was selected such that 0.45 < a/W < 0.55 with “a” identifying the initial crack length.

The fracture tests were performed by using a mechanical testing machine (Instron 3360 Dynamometer, Norwood, MA, USA) equipped with a 250 N load cell and by setting a quasi-static displacement rate of 1 mm/min. The value of the stress intensity factor is computed according to Equation (1), as:

(1) $$K_{\mathrm{IC}}=f\left(\frac{a}{W}\right)\left(\frac{P_{Q}}{{\mathrm{BW}}^{\frac{1}{2}}}\right)$$

with:

(2) $$f\left(\frac{a}{W}\right)=6{\left(\frac{a}{W}\right)}^{1/2}\frac{\left[1.99-\left(\frac{a}{W}\right)\left(1-\frac{a}{W}\right)\left(2.15-3.93\frac{a}{W}+2.7{\left(\frac{a}{W}\right)}^{2}\right)\right]}{\left(1+2\frac{a}{W}\right){\left(1-\frac{a}{W}\right)}^{3/2}}$$

and P_Q_ is the load at failure. Critical strain energy release rates (G_IC_) were calculated from the stress intensity values according to the standard. The fracture mechanism was analyzed using SEM observations of the SENB sample. Six separate measurements were performed to provide the average value and the deviation for each CSNPs’ composition.

## 3. Results and Discussion

### 3.1. CSNPs Characterization

**Figure 3:**
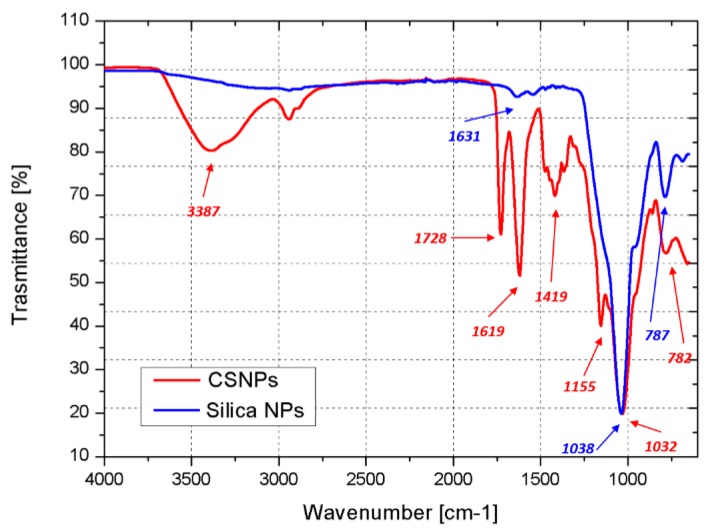
Fourier transform infrared (FTIR) spectra of functionalized silica nanoparticles (NPs) (blue curve) and CSNPs (red curve).

The FTIR spectra of functionalized silica NPs and CSNPs are shown in Figure 3. In the functionalized silica NPs spectrum (blue curve), the peaks at 787 and 1038 cm^−1^ are associated with the stretching of the Si–O and Si–OH bonds, respectively. The stretching of the amino group is centered at 1631 cm^−1^. In the spectrum of the CSNPs (red curve), the HBP growth on the silica surface is highlighted by the appearance of new peaks. In particular, the broad band at 3387 cm^−1^ is associated with the –OH stretching while the weak absorptions at 1155 and 1419 cm^−1^ correspond to the C–O stretching and the C–N bond, respectively. Strong peaks centered at 1619 and 1728 cm^−1^ are respectively associated with N–H stretching and –C=O stretching.

**Figure 4:**
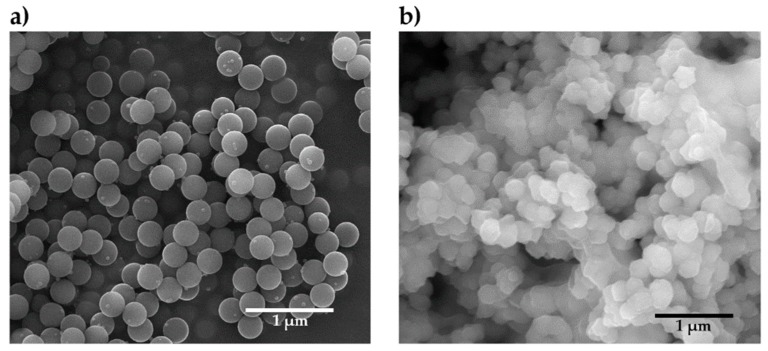
Scanning electron microscopy (SEM) micrographs of (**a**) functionalized silica NPs and (**b**) CSNPs.

**Figure 5:**
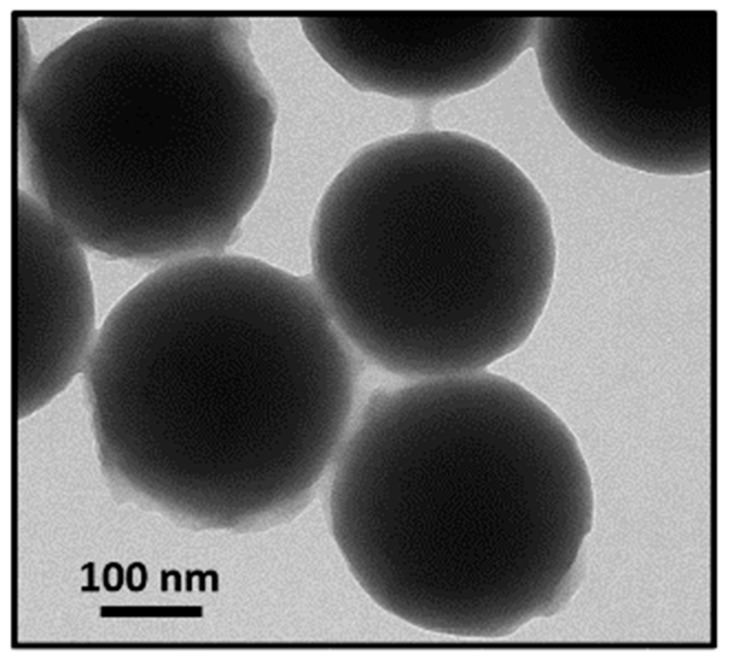
Transmission electron microscopy (TEM) micrographs of CSNPs.

In Figure 4, the SEM micrographs of both functionalized silica NPs (a) and CSNPs (b) are reported. For functionalized silica NPs, see Figure 4a, an average diameter of about 250 nm can be observed. Since no remarkable variation of the diameter is evidenced after grafting of the HBP in the CSNPs, this suggested the formation of a thin polymer shell surrounding the silica core of NPs, as confirmed by TEM images, see Figure 5.

The thickness of the HBP shell is equal to ~15 nm. It is noteworthy that CSNPs tend to slightly agglomerate due to the strong polarity of the hydroxyl and amino terminal groups.

**Figure 6:**
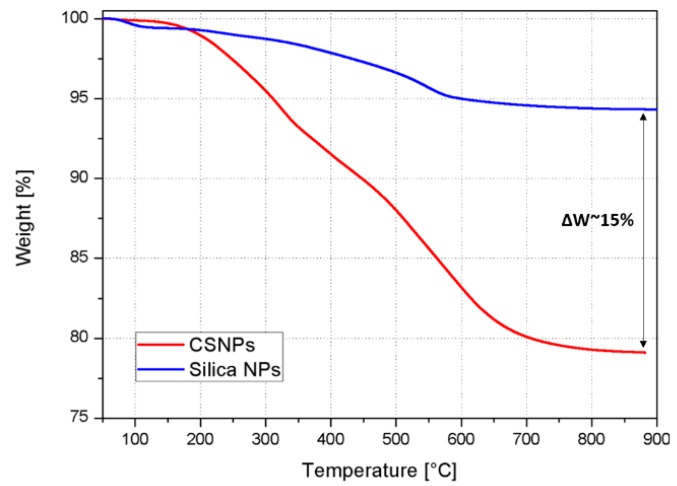
TGA thermograms (in nitrogen) of (blue curve) functionalized NPs and (red curve) CSNPs.

The presence of the polymeric shell in the CSNPs was also confirmed by TGA, see Figure 6. The minor residual weight at 900 °C of the CSNPs compared to the functionalized NPs (ΔW ~ 15% less) indicates the degradation of an organic phase, which is instead absent in the pristine silica NPs.

**Figure 7:**
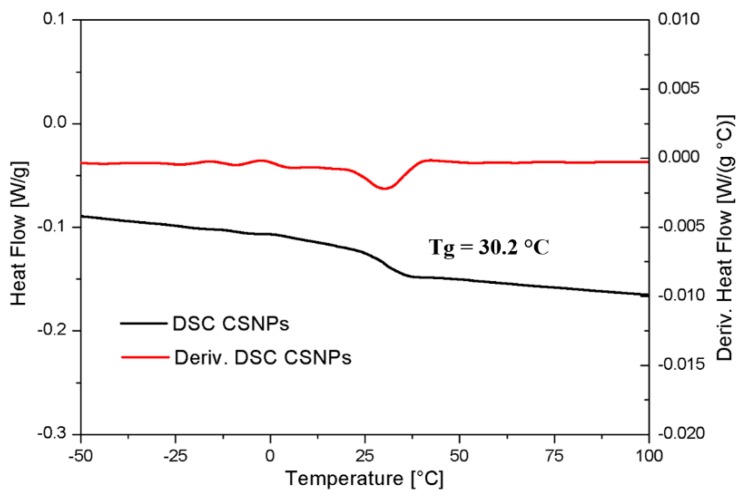
DSC and derivative differential scanning calorimetry curves of CSNPs.

By DSC analysis of CSNPs it was possible to estimate the T_g_ of the HBP shell, which was found to be ~30 °C, see Figure 7.

### 3.2. CSNPs/RTM6 Nanocomposites Characterization

#### 3.2.1. Dynamic Mechanical Properties

**Figure 8:**
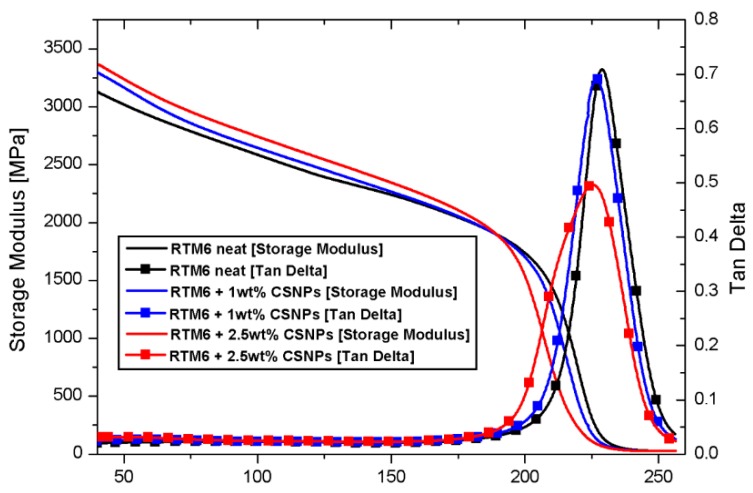
Dynamic mechanical analysis (DMA) curves of neat (black curve), 1 wt% (red), and 2.5 wt% (blue) filled systems.

The dynamic mechanical properties of the CSNPs/RTM6 nanocomposites were examined by DMA over a range of temperatures (40–260 °C). As reported in the DMA graph of Figure 8, the addition of CSNPs to the epoxy resin does not remarkably affect the thermomechanical properties of the hosting matrix, as suggested by a very negligible reduction of T_g_ of about ~4 °C. The value of T_g_ was derived by considering the temperature corresponding to the maximum of the tan δ curve.

The lowering of T_g_ could be related to the reduction of the matrix crosslink density and to the addition of a polymeric phase (i.e., the HBP shell) with a lower transition temperature. Moreover, the increase of the elastic modulus from 3107 MPa to 3392 MPa (approx. +10%) is attributable to the presence of an inorganic core of nanoparticles, which increases the nanocomposites’ stiffness by constraining the polymeric chains and limiting their movement [13]. Table 1 presents the DMA results.

#### 3.2.2. Thermal Stability

**Figure 9:**
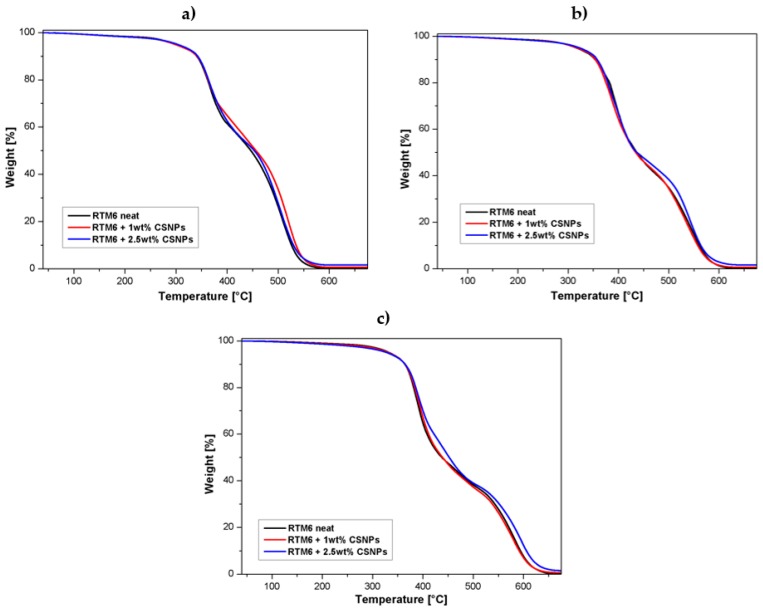
TGA curves (air atmosphere) of neat epoxy and nanocomposites at different heating rates: (**a**) 2 °C/min, (**b**) 5 °C/min and (**c**) 10 °C/min.

Figure 9 shows TGA curves relative to neat epoxy and CSNPs/RTM6 nanocomposites; these tests were performed in an oxidative atmosphere (air) using three different heating rates (2 °C/min, 5 °C/min, and 10 °C/min). The typical degradation behavior of the epoxy resins, characterized by two main changes in slopes of the curve, was recorded. These two steps can be explained as follows:

1. A first degradation step up to 450 °C, which mainly involves dehydration (around 100 °C) of the material and formation of a poly-aromatic structure [22];
2. A second step between 500–600 °C, characterized by thermo-oxidative reactions, which lead to the complete degradation of the carbonaceous materials and char formation [22].

It is noteworthy from Figure 9 that the addition of CSNPs does not induce any changes in the degradation curve shape, indicating that CSNPs act as an inert filler without any chemical interaction with the hosting system.

In Table 2, the TGA results of the tests performed in an oxidative atmosphere with a heating rate of 10 °C/min on neat and CSNP-loaded resins are reported. The end set of the thermal degradation temperature was determined from the intersection of the two tangents, while the T^Peak I^ and T^Peak II^ parameters were computed by the corresponding DTGA (Derivative TGA) curves as the temperatures associated to the maximum weight loss, respectively, for the first and second degradation step.

Dynamic TGA test results can be employed to determine the activation energies of the oxidation process by means of the Kissinger Method (KM) without any pre-assumption of the thermal degradation mechanism to analyze the TGA experimental data.

For dynamic TGA measurements, mass loss is monitored as a function of temperature at different heating rates and kinetics degradation, and in its general form, can be modelled as:

(3) $$\frac{d\alpha }{\mathrm{dt}}=f\;\left(\alpha ,\;T\right)$$

where T and α are the temperature and mass loss, respectively. Assuming an nth reaction order mechanism and considering an Arrhenius temperature dependence for the degradation rate, it can be written:

(4) $$\frac{d\alpha }{\mathrm{dt}}=K\left(T\right)f\left(\alpha \right)$$

where K can be expressed as follows:

(5) $$K=A\exp\left(-\frac{E}{\mathrm{RT}}\right)$$

where, A is a pre-exponential factor and E is the activation energy of the degradation step. Combining Equations (4) and (5):

(6) $$\frac{d\alpha }{\mathrm{dt}}=A\;f\left(\alpha \right)\exp\left(-\frac{E}{\mathrm{RT}}\right)$$

If the heating rate β = dT/dt is constant, the kinetics of degradation can be analyzed as a function of temperature:

(7) $$\frac{d\alpha }{\mathrm{dT}}=\frac{A}{\beta }\;f\left(\alpha \right)\exp\left(-\frac{E}{\mathrm{RT}}\right)$$

The integrated form of Equation (7) is generally expressed as:

(8) $$g\left(\alpha \right)=\int_{0}^{\alpha }\frac{d\left(\alpha \right)}{f\left(\alpha \right)}\;=\;\frac{A}{\beta }\int_{0}^{\alpha }\exp\left(-\frac{E}{\mathrm{RT}}\right)\;\mathrm{dT}\;$$

where g(α) is the integrated form of the conversion dependence function. The Kissinger Method is based on the following equation:

(9) $$\ln\left(\frac{\beta }{T_{\max}^{2}}\right)=\;\left\{\ln\frac{\mathrm{AR}}{E}+\ln\left[n\;{\left(1-{\;\alpha }_{\max}\right)}^{n-1}\right]\right\}-\;\frac{E}{{\mathrm{RT}}_{\max}}$$

which correlates to the changes in the heating rate, β, with the maximum peak temperatures, T_max_, obtained by DTGA curves. In our analysis: T_max_ corresponds to T^Peak I,II^, α_max_ is the conversion associated to T_max_ and n is the reaction order. Assuming that f’(α_max_) = n(1 − α_max_)^n−1^ ≈ const, it is possible to evaluate the activation energy E from a plot of ln(β/T^2^_max_) vs. 1/T_max_.

**Figure 10:**
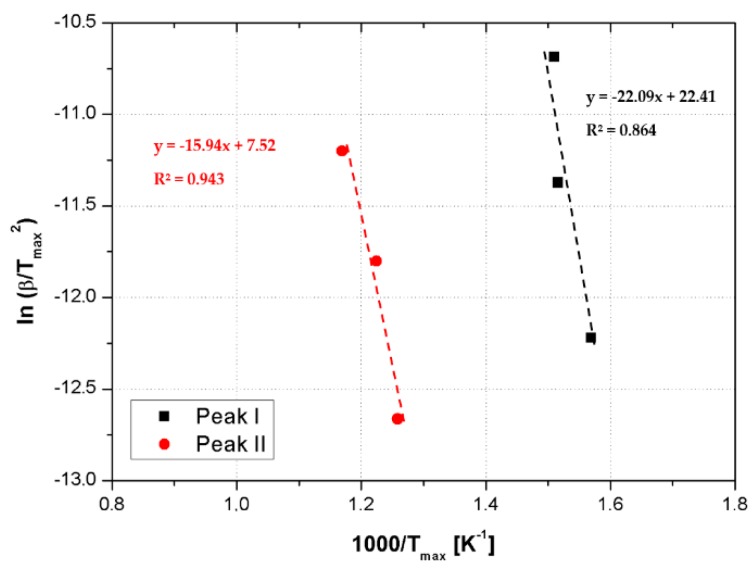
Kissinger Method applied to the RTM6 + 1 wt% CSNPs sample.

Therefore, the activation energies for each degradation step correspond to the slopes of the straight lines fitting of ln(β/T^2^_max_) vs. 1/T_max_, see Figure 10 for the sample RTM6 + 1 wt% CSNPs, according the following equation [23]:

(10) $$\ln\left(\frac{\beta }{T_{\max}^{2}}\right)=\;\ln\frac{\mathrm{AR}}{E}\;-\;\frac{E}{{\mathrm{RT}}_{\max}}$$

**Figure 11:**
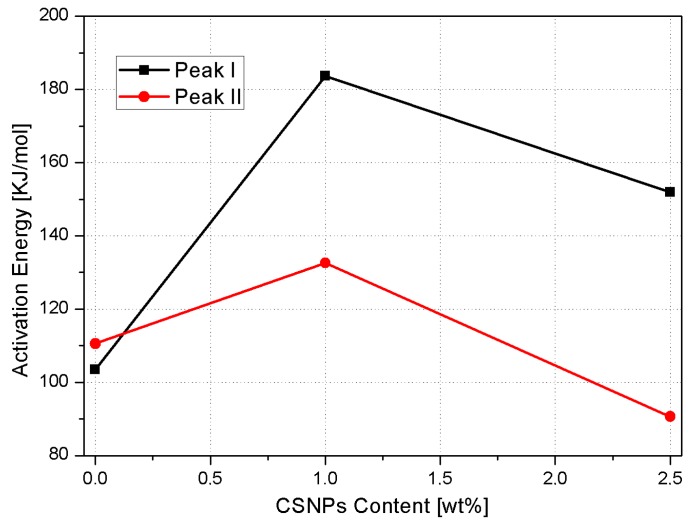
Activation energies as function of CSNPs content for both degradation steps.

Table 2 reports the Kissinger analysis results relative to neat epoxy and nanocomposites. The Kissinger model reveals a very interesting behavior, see Figure 11 and Table 3, of the nanocomposite systems, summarized as:

(1) At low CSNPs content (1 wt%), the activation energies of both degradation steps increased compared to the neat resin. This behavior can be explained assuming that CSNPs act as physical barriers both to postpone the thermal decomposition of volatile components and to prevent the transport phenomena of volatile decomposed products into the hosting matrix [24]. Therefore, at lower filler contents, the predominant effect associated with the presence of an inorganic core is an increase of the activation energy;
(2) Higher CSNPs content (2.5 wt%) induces a reduction of both activation energies: In accordance with Zhou et al. [25], the HBP shell of the CSNPs can induce a lowering of activation energies associated with the reduction of the crosslink density in the hosting matrix. The energy reduction effect associated with the presence of the polymeric shell became more relevant compared to the sample filled at 1 wt% of CSNPs.

#### 3.2.3. Fracture Toughness Results

Fracture tests were performed by using a three-point bending configuration according to the ASTM D5045 standard for a polymer system. Typical linear load–displacement curves have been obtained for the neat epoxy and its nanocomposites, both showing a brittle fracture behavior. The stress intensity factor represents the constant of proportionality between the applied stress and square root of the distance from the crack and it is used to determine the fracture toughness of most materials. Because the dependence of the stresses on the coordinate variables remains the same for different types of cracks and shaped bodies, the stress intensity factor is a single parameter characterizing the crack tip stress field. The value of the stress intensity factor at which unstable crack propagation occurs is called the fracture toughness or critical stress intensity factor, K_IC_. Maximum loads are used to calculate the fracture toughness of the samples along with the initial crack length.

**Figure 12:**
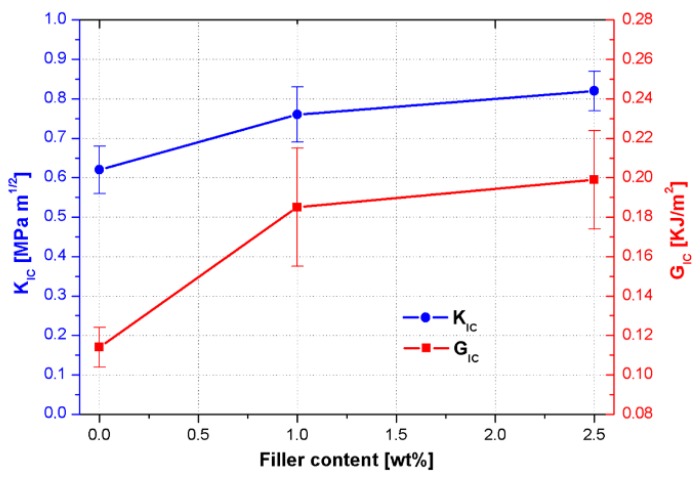
K_IC_ and G_IC_ behaviors obtained from fracture toughness tests for neat and filled systems.

Figure 12 displays the effect of CSNPs on the fracture toughness of CSNPs/RTM6 systems and the corresponding data are reported in Table 4. It is clear that both K_IC_ and G_IC_ increase with the addition of CSNPs; it could be found that, when the content of CSNPs is 2.5 wt%, the K_IC_ value is improved to 0.82 MPa m^1/2^ from 0.62 MPa m^1/2^ for the neat resin, which corresponds to an increase of 32%.

For the sample filled with 2.5 wt% of CSNPs, a higher increase is obtained of the G_IC_ value, which rises from 0.114 to 0.199 KJ/m^2^, recording a positive variation of about +75%.

The improvement of the fracture behavior could be attributable to:

- reduction of the crosslink density which affects the internal stress level induced during the epoxy cure [26];
- induction of a triaxial stress state in the matrix around CSNPs due to the presence of the inorganic core and thus promoting the plastic deformation mechanism in the polymeric phase and then crack tip blunting [27];
- further promotion of plastic deformation associated with the presence of the HBP shell [28].

**Figure 13:**
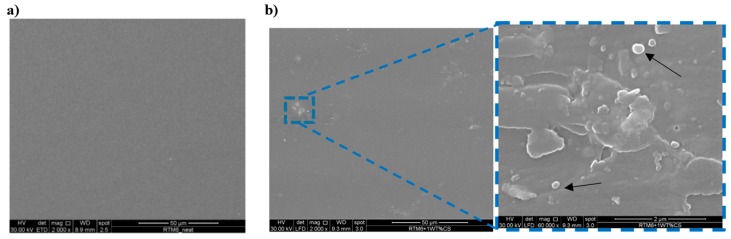
SEM micrographics of (**a**) neat epoxy resin and (**b**) RTM6 + 1 wt% CSNPs: the zoom in (**b**) clearly shows the CSNPs (black arrows).

In order to evaluate these micro-mechanical mechanisms leading to an improvement of the fracture toughness performances, the fractured surfaces of the nanocomposite samples were observed using SEM images, see Figure 13. In Figure 13a, the neat epoxy shows a very smooth fractured surface due to the intrinsic brittleness of the epoxy matrix. This fractography is typical for brittle polymers and it reveals that the crack propagation is fast and catastrophic. Comparing the SEM fractographic images of nanofilled samples, see Figure 13b, it results that the fracture surface, although still brittle, presents a high surface roughness associated with the presence of large plastic deformation areas, and the presence of CSNPs is highlighted by analyzing the picture magnification (black arrows). In fact, due to the absence of crack pinning tails (typical of the epoxy resins loaded with rigid particles without a soft shell [29]), the main source of energy dissipation during nanocomposites fracture is the plastic deformation of the matrix surrounding the CSNPs.

## 4. Conclusions

The influence of synthesized silica core/HBP shell NPs on the epoxy resin thermal stability and fracture toughness has been investigated, and the results are presented and analyzed. FTIR analysis reveals the existence of the HBP in the CSNPs samples because the presence of peaks, absent in the neat silica NPs spectrum, could be related to the polymer functional groups characterizing the surrounding shell. These observations are in agreement with the TEM micrographs, which indicate the presence of a thin polymeric shell, which encapsulate the silica NPs.

Thermomechanical results confirm that addition of CSNPs to the epoxy matrix induces a slight reduction of T_g_ (~4 °C) and an increase of the storage modulus of about ~10%. Thermal stability was studied by TGA analysis, and data were modeled by employing the Kissinger Method; the results indicated an increase of degradation activation energies with low filler content (1 wt%), and that further addition of CSNPs (2.5 wt%) induces a reduction of these activation energies. Final data for CSNPs/epoxy systems indicate that toughness enhancement occurred showing thatK_IC_ and G_IC_ increase of 32% and 74%, respectively. SEM observation of the fracture surfaces reveals that the main source of energy dissipation during crack propagation is the plastic deformation of the hosting matrix.

## Figures and Tables

**Table 1:** Glass transition temperature (T_g_) and storage modulus of the CSNPs/RTM6 nanocomposites obtained by DMA.

Sample	T_g_ [°C]	Storage Modulus at 40 °C [MPa]
**Neat RTM6**	228.9	3107 ± 15
**RTM6 + 1 wt% CSNPs**	226.8	3296 ± 21
**RTM6 + 2.5 wt% CSNPs**	225.4	3392 ± 23

**Table 2:** Results of TGA tests (in air) for CSNPs/RTM6 systems.

Sample	T^end set I^ (°C)	T^end set II^ (°C)	T^Peak I^ (°C)	T^Peak II^ (°C)	Char Yield (%)
**Neat RTM6**	407.9	612.7	387.0	584.1	0.06
**RTM6 + 1 wt% CSNPs**	408.8	611.1	389.0	581.9	0.73
**RTM6 + 2.5 wt% CSNPs**	406.7	621.8	393.0	595.9	1.61

**Table 3:** Kissinger activation energies for each of the degradation steps for the CSNPs/RTM6 systems in air.

Sample	E_a_^I^ (KJ/mol)	E_a_^II^ (KJ/mol)
**Neat RTM6**	103.5	110.6
**RTM6 + 1 wt% CSNPs**	183.7	132.6
**RTM6 + 2.5 wt% CSNPs**	152.0	90.6

**Table 4:** Effect of CSNPs content on the nanocomposites fracture toughness.

Sample	K_IC_ [MPa m^1/2^]	ΔK_IC_	% Variation K_IC_	G_IC_ [KJ/m^2^]	ΔG_IC_	% Variation G_IC_
**RTM6 Neat**	0.62	0.06	-	0.114	0.01	-
**RTM6 + 1 wt% CSNPs**	0.76	0.07	22.6	0.185	0.03	62.3
**RTM6 + 2.5 wt% CSNPs**	0.82	0.05	32.3	0.199	0.025	74.6
